# Proboscis Morphology and Its Relationship to Feeding Habits in Noctuid Moths

**DOI:** 10.1673/031.011.0142

**Published:** 2011-04-06

**Authors:** Maurício Moraes Zenker, Carla Penz, Michele de Paris, Alexandre Specht

**Affiliations:** ^1^ Laboratório de Entomologia, Departamento de Biodiversidade e Ecologia, Faculdade de Biociências, Pontifícia Universidade Católica do Rio Grande do Sul. Av. Ipiranga, 6681, 90619-900 Porto Alegre, RS, Brazil; ^2^ Department of Biological Sciences, University of New Orleans, New Orleans, LA, 70148, USA.; ^3^ Laboratório de Biología, Centro de Ciências Exatas, da Natureza e de Tecnologia, Campus Universitário da Região dos Vinhedos, Universidade de Caxias do Sul, Caixa Postal 32, 95700-000 Bento Gonçalves, RS, Brazil; ^4^ Instituto de Biotecnologia, Centro de Ciências Agrárias e Biológicas, Universidade de Caxias do Sul, Cidade Universitária. Caixa Postal 1352, 95070-560, Caxias do Sul, RS, Brazil

**Keywords:** fruit-piercing moths, morphometrics, Calpinae, South America

## Abstract

This study describes proboscis morphology and identifies morphometric differences among five species of noctuid moths with different feeding habits (fruit versus nectar-feeding). Morphological and morphometric parameters were analyzed using scanning electron microscopy and light microscopy. Measurements included: galea height in ten sites from base to tip, total proboscis length, and length of the distal region that contains large sensilla styloconica and / or tearing hooks and erectible barbs. Both morphometric and morphological differences were identified among species within and between feeding guilds, and these results are discussed in light of the feeding habits of each species.

## Introduction

Although most adult Lepidoptera of the suborder Glossata visit flowers to feed on nectar, specialized feeding habits have evolved independently in various groups (see Krenn 2010 for a review). Most non-flower-visiting moths and butterflies feed on exposed surfaces that may include the nitrogen-rich juice of decaying fruit, extra-floral nectar, sap, or animal fluids (Büttiker 1967; Büttiker et al. 1996; Bänziger 1970; Krenn et al. 2001; Molleman et al. 2005). Some species, however, are capable of piercing the substrate to gain access to the internal flesh of either plant or animal origin (e.g., Büttiker et al. 1996; Zaspel et al. 2007). The parallel evolution of fruit-feeding led to morphological modifications of the proboscis that are convergent between distantly related families of Lepidoptera (Krenn 2010).

The first broad-scale study that investigated the correlation between proboscis morphology and feeding habits was Krenn et al. (2001). By comparing flower-visiting and non-flower-visiting nymphalid butterflies (64 species), the authors identified proboscis modifications that evolved independently in various butterfly subfamilies and reflected adaptations to novel food resources (decaying fruit or sap). Interestingly, that study showed that proboscis attributes in non-flower-visiting butterflies were similar to those described for non-flower-visiting noctuid moths which feed from the surface and are not capable of piercing (e.g., Bänziger 1970; Büttiker et al. 1996).

Species of the family Noctuidae vary in feeding habits and proboscis morphology (Büttiker 1967; Bänziger 1970; Hendrix et al. 1987; Lingren et al. 1993). The proboscis of nectivorous species is characterized by a relatively simple tip region devoid of ‘spines’ and with few sensilla (Bänziger 1970). In contrast, fruit-piercing moths in the Calpinae and some of the Catocalinae can lacerate the pulp of damaged fruits or even pierce the intact rind (secondary and primary fruitpiercing *sensu* Bänziger 1982). Several *Calyptra* (Calpinae) feed on blood and are, therefore, capable of puncturing mammal skin (Bänziger 1989; Zaspel et al. 2007). The probosces of non-flower-visiting noctuids are typically armed with various long ‘spines’ and bear numerous sensilla at the tip region (e.g., Bänziger 1970). The diversity of feeding habits within Noctuidae makes this family an interesting focal group for studies of proboscis morphology.

Fruit-piercing noctuid moths are known to cause considerable damage to cultivated citrus crops (Fay and Halfpapp 2006), and some species feed on grapes in northeastern Brazil (Haji et al. 2001). Given the potential impact of fruit-piercing moths on fruits crops, baseline studies of their feeding habits, morphology, and natural history are important. Therefore the aims of this study are to characterize Neotropical fruit-feeding noctuid moths and compare their proboscis morphology to nectivorous species occurring in the same area. To do so we examined the proboscis of five species in four subfamilies with optical and scanning microscopy to determine if fruit-feeding species possessed correlated morphological characteristics. Our results indicate that two of our focal species possess attributes previously described for fruit-piercing moths, therefore contributing to the understanding of a neotropical moth fauna within agricultural ecosystems.

## Materials and Methods

### Species sampled

The following species of Noctuidae were examined (classification follows Fibiger & Lafontaine 2005): *Alabama argillacea* (Hübner, 1823) and *Gonodonta bidens* Geyer (both Calpinae); *Rachiplusia nu* (Guenée) (Plusiinae); *Mods latipes* Hübner (Catocalinae); and *Chabuata major* (Guenée) (Hadeninae). These species were selected because fruit-feeding habits were known for *A. argillacea* (Costa Lima 1950) and the genus *Gonodonta* (Todd 1959), yet their proboscis morphology had not been examined in detail. Bänziger (1982) noted that two species of *Mocis* in Thailand are capable of piercing soft-skinned fruit, although European Catocalinae feed on tree sap (H. Krenn pers. comm.). Except for *R. nu*, all species had similar forewing length, used here as a proxy for body size.

Study specimens were obtained from light and pheromone traps, and museum collections (Museu de Ciência e Tecnologia, Pontifícia Universidade Católica do Rio Grande do Sul, and Laboratório de Biologia, Universidade de Caxias do Sul, Brazil). Although an equal number of males and females were examined per species, sample size varied among species (Tables 1 and 2).

**Table 1.**
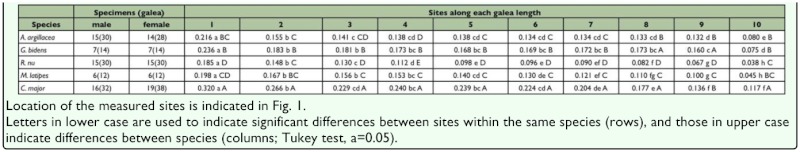
Mean height (mm) of ten proboscis sites for five Noctuidae species; number of male and female examined, and number of galeae measured are given for each species.

**Table 2.**
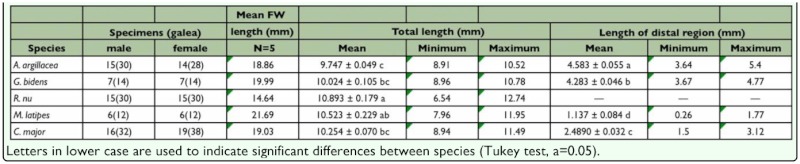
Measurements of forewing length (FW), total proboscis length and range, length of the distal region that bears sensilla and range.

### Light microscopy

The entire proboscis of each specimen was removed from the head and submerged in lactic acid 50% until the galeae were separated from each other (see Krenn et al. 2001 for protocol). Each individual galea was embedded in Entellan (www.merckchemicals.com), mounted on microscope slides with the lateral portion facing up, and sealed with a coverslip. Slides were labeled with the species name, sex, and a voucher number. The prepared slides were deposited as a lot at Universidade de Caxias do Sul, Brazil (CUCS accession number 01-240).

**Figure 1.**
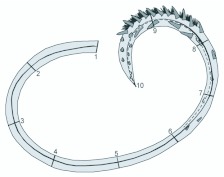
Schematic representation of the proboscis showing the measured sites I (base) to 10 (tip). Black line represents the total length, and dashed line indicates the region that bears sensillae and tearing hooks/erectible barbs. High quality figures are available online.

For morphometric analyses each galea was photographed with a digital camera attached to a stereomicroscope at magnifications 10, 12, 16, 20, 25, 32 and 66 X. Digital photographs were used for measuring ten variables as indicated in Figure 1. We used the measurement software AxioVision Rel. 4.1 (Carl Zeiss, www.zeiss.com) calibrated with digital caliper rule for the 10, 12 and 16 X magnifications, and with a micrometric slide for the 20, 25, 32 and 66X magnifications. Multiple magnifications were used for the measurements, and repeatability of measurements with different magnifications attested to their overall accuracy. Both galeae of each specimen were measured, and the two sets of values obtained were entered in the statistical analyses. This was done to reduce possible effects of preparation artifacts.

Twelve parameters were measured in each galea (Figure 1): total length; galea height at ten evenly spaced intervals along its length, including the base and tip; length of the ‘distal region’, defined here as the portion of the proboscis that bears rasping spines and sensilla (except for *R. nu*, that had minute sensilla which could not be clearly visualized in the photographs). Fruit-piercing noctuids are known to have a short proboscis of nearly uniform width along its length, with robust galeae holding large bundles of muscles (Bänziger 1970). Therefore, the ‘absolute height’ and ‘evenness of height’ along the length of the galea were used as a proxy for ‘general robustness’.

### Scanning electron microscopy

For each species an individual galea of each sex was examined with a Philips XL 30 Scanning Electron Microscope (SEM) to analyze the surface microstructure of the distal region of the proboscis and prepare images (Center of Microscopy and Microanalysis, Pontifícia Universidade Católica-RS). The base of the proboscis and the food canal were not examined with SEM, but are known to contain sensilla trichodea and basiconica (Krenn 2010). The material was dehydrated in alcohol 100% for 15 min, followed by 15 min in acetone, then a critical point dryer Bal-Tec CPD 030 was used to remove all moisture from the samples (Castro 2002). Each galea was mounted on an aluminum stub with a graphite adhesive double-face tape, and sputter-coated with gold and carbon using a Bal-Tec SCD 005 unit.

**Figure 2.**
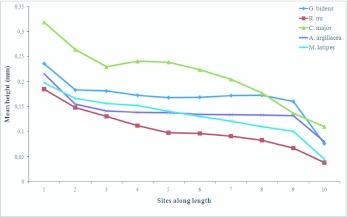
Mean width in ten sites along galea total length showing differential decline in width for five Noctuidae species. High quality figures are available online.

**Figure 3.**
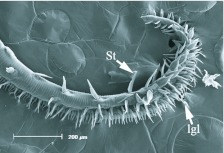
Distal region of the proboscis *of Alabama argillacea*. St, erectible sensilla styloconica; lgl, legulae of the galea linkage (rasping spines of Bänziger 1970). High quality figures are available online.

**Figure 4.**
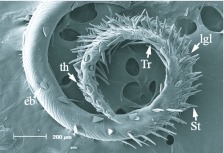
Distal region of the proboscis of *Gonodonta bidens*. St, erectible sensilla styloconica; Tr, sensilla trichodea; lgl, legulae of the galea linkage (rasping spines of Bänziger 1970); th, tearing hooks; eb, erectible barbs. High quality figures are available online.

**Figure 5.**
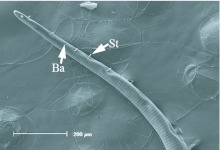
Distal region of the proboscis of *Rachiplusia nu*. St, sensilla styloconica; Ba, sensilla basiconica. High quality figures are available online.

### Data analysis

An analysis of variance was used to compare measurements within and between species (ANOVA plus Tukey test at α = 0.05; SPSS 14.0 software). Species, sexes and ten sites along the proboscis were used as factors; total length, length of the tip and the height of each of the ten sites were considered as dependent variables. This allowed us to evaluate and compare: (a) differences between the sexes, (b) variation in height among ten galea segments within species, (c) variation in height among galea segments between species, (d) variation in proboscis length between species, and (e) variation in length of the distal region between species.

## Results

As males and females within species did not differ in any parameters measured (results not shown) the sexes were pooled for comparisons between species (Tables 1 and 2, Figure 2). It is verified here for the first time that the galea of fruit-piercing moths has an even height along its length, and that this proboscis attribute differs between fruitpiercing and nectivore species. Mean height was not uniform along the length of the proboscis within species, with the base being taller than the tip (Table 1). All species showed a significant decrease in galea height between sites 1–2 and, except for *C. major*, between sites 9–10. Nonetheless, the rate of decrease from base to tip differed among species (Table 1, Figure 2). In fruit-piercing *A. argillacea* and *C. major* the galea height also decreased significantly between sites 2–3, after which height decreased rather gradually along the proboscis length with a sharp decrease between sites 9–10. Although a steady decrease in height was also observed in nectar feeding species, significant decreases in height were observed between sites 8-9-10 in *R. nu* and *C. major*. An unusual enlargement was found between sites 3–4 in *C. major*, which was maintained in sites 4–5. The galea of *M. latipes* showed the most even decrease in height among the three studied nectivores. These results showed that the rate in which the galea decreased in height from base to tip varied slightly within Calpinae, and more strongly between fruit-piercing and nectivores.

**Figure 6.**
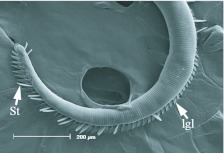
Distal region of the proboscis of *Mods latipes*. St, sensilla styloconica; lgl, legulae of the galea linkage (rasping spines of Bänziger 1970). High quality figures are available online.

**Figure 7.**
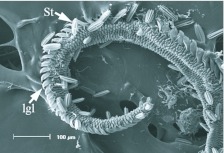
Distal region of the proboscis of *Chabuata major*. St, pluricarinate sensilla styloconica; lgl, legulae of the galea linkage (rasping spines of Bänziger 1970). High quality figures are available online.

Galea height varied between species (Table 1). Despite the small difference in wing length (Table 2), the galea of the fruit-piercing *A*. *argillacea* and *G. bidens* differed in height at all sites except 1 and 10. The nectivore *C. major* had a significantly taller galea than all other species at both the base and tip, and along the proboscis length (i.e., a more robust overall aspect). Finally, given the small galea height, the proboscis of *R. nu* was clearly the most delicate among all five species.

Both total proboscis length, and length of the distal region varied between species (Table 2). The proboscis of nectivores was slightly longer than that of fruit-feeding *A. argillacea* and *G. bidens*. On average *R. nu* had the longest proboscis, and *A. argillacea* the shortest. The length of the distal region bearing sensilla and/or spines differed significantly among the four species, including the fruit-piercing *A. argillacea* and *G. bidens*. However, the length of the distal region was similar in *A. argillacea* and *G. bidens*, and much longer than in the nectivores *C. major* and *M. latipes* (approximately 2x and 3x longer, respectively).

The general morphology of the distal region varied among the five species, with the two fruit-piercing calpines most similar to each other (Figure 3–7). The dorsal legulae of the galeal linkage (terminology from Büttiker et al. 1996; ‘rasping spines’ in Bänziger 1970) are spine-like in *A. argillacea* (Figure 3) and *G. bidens* (Figure 4), being longer in *A. argillacea* than in *G. bidens*. In contrast, they are broad in flower-visiting species (Figure 5–7). *Alabama argillacea* and *G. bidens* had a larger number of erectible sensilla styloconica on the distal region compared to *R. nu* (Figure 5) and *M. latipes* (Figure 6). The distal proboscis of fruit-piercing *G. bidens* was more complex than *A. argillacea*. In addition to sensilla styloconica, Figure 4 shows that *G. bidens* also has sensilla trichodea, ‘tearing hooks’ and ‘erectible barbs’ (terminology from Bänziger 1970). Interestingly, the proboscis of flower-visiting *C. major* (Figure 7) is heavily textured and has many pluricarinate sensilla styloconica (terminology from Petr & Stewart 2004).

## Discussion

The independent evolution of fruit-feeding in Lepidoptera produced interesting modifications of the proboscis (e.g., Noctuidae and Nymphalidae; Büttiker 1967; Bänziger 1970; Krenn et al. 2001; Krenn 2010). Within Noctuidae, members of Calpinae have proboscis and behavioral adaptations that enable them to pierce fruit skin, and fruit-feeding seems to have preceded the remarkable habit of using animal blood and tears as a nutritional resources (Büttiker 1967; Büttiker et al. 1996; Bänziger 1970, 2007; Hilgartner et al. 2007). Such work provided the impetus for our study, particularly in light of the potential negative impact of fruit-piercing moths on agricultural crops (see Haji et al. 2001).

The volume of muscle bundles inside the galeal lumen affects both the width and height of the galea (see Bänziger 1970 and Krenn 2000 for cross-sections). Given that fruitpiercing species forcefully penetrate the substrate upon which they feed, their proboscis is likely to be more robust than that of nectivores. Galea height was more evenly maintained along the proboscis length in fruitpiercing than in nectivore species where the decline in height from base to tip was more pronounced (Table 1). This result complements previous studies showing a small decline in proboscis width in fruitpiercing moths (Bänziger 1970), and suggests larger muscle bundles are needed in fruitpiercing species. We found no significant differences in mean galea height between species in fruit-piercing and nectar-feeding guilds as predicted by Bänziger (1970). For most of the proboscis length (segments 2–8), galea height was significantly different among the five species suggesting that our sample size was too small to separate interspecific differences from those attributed to feeding habits. Despite similarity in proboscis length (Table 2), *G. bidens* had a significantly more robust proboscis than *A. argillacea*, thus suggesting *G. bidens* may be more efficient at piercing fruit than *A. argillacea*.

Previous studies suggest that the proboscis of fruit-feeding species is generally shorter than nectivores (e.g., Bänziger 1970). Indeed, a long proboscis is not needed for feeding on exposed surfaces, and natural selection likely favored a reduction of proboscis length in fruit-feeding nymphalids (Krenn et al. 2001). Although our sample size was small, we found no significant differences in proboscis length between fruit and nectar-feeding noctuids. Unlike moths that feed on exposed surfaces (sap or damaged fruit), fruit-piercing species must use force to penetrate the fruit rind and also have to reach into the fruit for feeding. Therefore, within Noctuidae we would expect to find species with shorter proboscis that fed on exposed surface (secondary fruit piercing), primary fruitpiercing species with moderately long proboscis, and nectar-feeding species with generally longer proboscis that might be associated with the type flowers they visit (e.g., Whittall and Hodges 2007). We found that neither galea height, nor length alone were good predictors of feeding habits in noctuid moths. A more comprehensive study would be required to compare statistically the proboscis length of primary and secondary fruit-piercing noctuids with that of nectivores.

In butterflies and moths the proboscis tip shows a number of morphological adaptations to fruit-feeding habits, and they differ in surface-feeding species and piercing species (Krenn 2010). In his study of Malaysian calpines (*Calyptra* and *Scoliopteryx*), Bänziger (1970) provided descriptions of proboscis morphology and fruit piercing behavior. We identified similar morphological characteristics in South American *A. argillacea* and *G. bidens*, such as long and spine-like dorsal legulae of the galeal linkage (Figure 3–4). While the proboscis distal region of both of the fruit-piercing species studied bear a large number of erectible sensilla styloconica, *G. bidens* also has sensilla trichodea, tearing hooks and erectible barbs. These differences strongly suggest that *A. argillacea* and *G. bidens* vary in their fruitpiercing capability, and may target different types of fruit, or fruit condition (intact versus damaged). Although the presence of specialized structures at the proboscis tip is a good predictor of feeding habits, variation in composition of these structures is known among fruit-feeding calpines (e.g., Bänziger 1970; Büttiker et al. 1996). Indeed, based on the proboscis tip morphology Hilgartner et al. (2007) suggested that Malagasy *Hemiceratoides hieroglyphica*, which feeds at eyes of sleeping birds, seem to be more closely related to fruit-feeding species than tear-drinking moths associated with mammals. The absence or presence of sensilla trichodea, tearing hooks and erectible barbs between *A. argillacea* and *G. bidens* may be due to phylogenetic relationships within Noctuidae, but further interpretation of feeding habits and morphological evolution requires more comprehensive sampling and a better resolution of noctuid phylogeny.

We found that *C. major* (Hadeninae) had characteristics of nectar-feeding species, such as broad galeal legulae, and the sensilla styloconica were noticeably more numerous in this species than in *R. nu* and *M. latipes*. Therefore, direct observations on *C. major* feeding habits would be of interest to determine if it opportunistically exploits resources other than nectar. Given that we found an unusual enlargement of *C. major* proboscis in sites 4–5, observations of proboscis movement would allow us to pinpoint the location of the usual ‘proboscis bend’ and verify whether that actually corresponds to a constriction in the measured site 3 (see Krenn 2010 for a discussion of the Glossata proboscis groundplan).

All available evidence suggests that the distinctive morphology of the tip region can be used to predict the feeding habits of noctuid species for which behavior and natural history information is unavailable. Given the broad distribution of Calpinae, this becomes particularly useful in areas where fruit-crops are being grown (see Bänziger 1982 for a brief overview). Together with knowledge of noctuid classification, examination of collection specimens should allow us to predict the number of potential fruit-piercing moths that occur in any given area. Such information is useful from the standpoint of agricultural entomology, and it will also provide insight on the ecology of noctuid feeding guilds in Subtropical South America.
